# Gut microbiota in experimental murine model of Graves’ orbitopathy established in different environments may modulate clinical presentation of disease

**DOI:** 10.1186/s40168-018-0478-4

**Published:** 2018-05-25

**Authors:** Giulia Masetti, Sajad Moshkelgosha, Hedda-Luise Köhling, Danila Covelli, Jasvinder Paul Banga, Utta Berchner-Pfannschmidt, Mareike Horstmann, Salvador Diaz-Cano, Gina-Eva Goertz, Sue Plummer, Anja Eckstein, Marian Ludgate, Filippo Biscarini, Julian Roberto Marchesi, the INDIGO consortium

**Affiliations:** 1 Division of Infection & Immunity, School of Medicine, Cardiff University, UHW main building, Heath Park, Cardiff, CF14 4XW UK; 2 Departments of Bioinformatics, PTP Science Park Srl, via Einstein loc. Cascina Codazza, 29600 Lodi, Italy; 3 Molecular Ophthalmology, Department of Ophthalmology, University Hospital Essen/University of Duisburg-Essen, 45147 Essen, Germany; 4 Faculty of Life Sciences and Medicine, King’s College London, London, SE5 9NU UK; 5 Latner Thoracic Surgery Laboratories, Toronto General Research Institute, University Health Network and University of Toronto, Toronto, M5G 1L7 Canada; 6 Cultech Ltd., Baglan, Port Talbot, SA127BZ UK; 7 University Hospital Essen, University of Duisburg-Essen, Institute of Medical Microbiology, 45147 Essen, Germany; 8 Graves’ Orbitopathy Center, Endocrinology, Department of Clinical Sciences and Community Health, Fondazione Ca’Granda IRCCS, University of Milan, via Sforza 35, 20122 Milan, Italy; 9 King’s College Hospital NHS Foundation Trust (SDC), London, SE5 9RS UK; 10 Italian National Council for Research (CNR), via Bassini 15, 20133 Milan, Italy; 11 School of Biosciences, Cardiff University, Sir Martin Evans Building, Museum Avenue, Cardiff, CF10 3AX UK; 12 Center for Digestive and Gut Health, Imperial College London, W2 1NY, London, UK; 13 INDIGO Consortium, http://www.indigo-iapp.eu

**Keywords:** Graves’ orbitopathy, Graves’ disease, Induced animal model, Gut microbiota, TSHR, Metataxonomics, Orbital adipogenesis, *Firmicutes*

## Abstract

**Background:**

Variation in induced models of autoimmunity has been attributed to the housing environment and its effect on the gut microbiota. In Graves’ disease (GD), autoantibodies to the thyrotropin receptor (TSHR) cause autoimmune hyperthyroidism. Many GD patients develop Graves’ orbitopathy or ophthalmopathy (GO) characterized by orbital tissue remodeling including adipogenesis. Murine models of GD/GO would help delineate pathogenetic mechanisms, and although several have been reported, most lack reproducibility. A model comprising immunization of female BALBc mice with a TSHR expression plasmid using in vivo electroporation was reproduced in two independent laboratories. Similar orbital disease was induced in both centers, but differences were apparent (e.g., hyperthyroidism in Center 1 but not Center 2). We hypothesized a role for the gut microbiota influencing the outcome and reproducibility of induced GO.

**Results:**

We combined metataxonomics (16S rRNA gene sequencing) and traditional microbial culture of the intestinal contents from the GO murine model, to analyze the gut microbiota in the two centers. We observed significant differences in alpha and beta diversity and in the taxonomic profiles, e.g., operational taxonomic units (OTUs) from the genus *Lactobacillus* were more abundant in Center 2, and *Bacteroides* and *Bifidobacterium* counts were more abundant in Center 1 where we also observed a negative correlation between the OTUs of the genus *Intestinimonas* and TSHR autoantibodies. Traditional microbiology largely confirmed the metataxonomics data and indicated significantly higher yeast counts in Center 1 TSHR-immunized mice. We also compared the gut microbiota between immunization groups within Center 2, comprising the TSHR- or βgal control-immunized mice and naïve untreated mice. We observed a shift of the TSHR-immunized mice bacterial communities described by the beta diversity weighted Unifrac. Furthermore, we observed a significant positive correlation between the presence of *Firmicutes* and orbital-adipogenesis specifically in TSHR-immunized mice.

**Conclusions:**

The significant differences observed in microbiota composition from BALBc mice undergoing the same immunization protocol in comparable specific-pathogen-free (SPF) units in different centers support a role for the gut microbiota in modulating the induced response. The gut microbiota might also contribute to the heterogeneity of induced response since we report potential disease-associated microbial taxonomies and correlation with ocular disease.

**Electronic supplementary material:**

The online version of this article (10.1186/s40168-018-0478-4) contains supplementary material, which is available to authorized users.

## Background

The poor reproducibility of murine models of human diseases has become a puzzling phenomenon in recent decades. Apart from the genetic background of the strains used, the type of animal housing, diet, and even the vendor can influence disease phenotype [1, 2].

In Graves’ disease (GD) and Graves’ orbitopathy or ophthalmopathy (GO), in vivo models could help to unravel the pathogenetic mechanisms leading to autoimmunity and identify new therapeutic targets [3]. The lack of spontaneous models of GD and GO necessitates induction of disease under laboratory conditions (reviewed in [4]).

Graves’ disease is an organ-specific antibody-mediated autoimmune disease, governed by both genetic predisposition and environmental factors, in which thyroid-stimulating antibodies (TSAb) mimic the function of thyroid-stimulating hormone (TSH) to activate the thyrotropin receptor (TSHR). Moreover, the presence of thyroid-stimulating blocking antibodies (TSBAb), which inhibit the TSHR signaling cascade, and neutral antibodies to TSHR have been described in GD [5]. GD symptoms include hyperthyroidism, weight loss, heat intolerance, and tremors; it affects about 2% of the population in the UK, with a female predominance. About 20% of GD patients develop an eye disease, GO, characterized by pro-inflammatory cells and tissue remodeling (extraocular muscle inflammation, adipogenesis, overproduction of extra-cellular matrix) in the orbit [6].

Several GD mouse models have been developed using different immunization protocols with no signs of concomitant eye disease as previously reviewed [4, 7, 8]. Ludgate and colleagues established a TSHR-induced GO model by genetic immunization, i.e., injecting a TSHR expression plasmid [9]. Mice developed thyroiditis plus some aspects of GO and disease could be transferred to naive recipients using the TSHR-primed T cells from the genetically immunized mice. However, the model could not be reproduced in a different animal unit (neither was specific-pathogen-free (SPF)), and the TSHR-induced disease was quite distinct from that previously described, which the authors postulated might be due to microorganisms [10]. It has also been reported that TSHR-immunized mice from a conventional environment had higher and more persistent TSAb levels than mice in SPF units [11].

Recently, Berchner-Pfannschmidt and colleagues reported the induction of GO-like disease in two independent SPF units [12]. The immunization protocol utilized genetic delivery of TSHR A-subunit plasmid by close field electroporation, which leads to features of GD accompanied by symptoms of eye disease, such as adipogenesis and inflammatory infiltrates in the orbit [7, 13]. Controls received a plasmid encoding the β-galactosidase (βgal) gene delivered by the same procedure. Most aspects of the model were reproduced successfully; however, there was heterogeneity in induced disease and differences in thyroid function in the animals undergoing experimental GO in the two locations [12].

Over the years, the gut microbiota has been associated with several diseases [14–17] and its confounding role in establishing or reproducing disease phenotype in murine models has also been proposed [18].

The murine model of multiple sclerosis, experimental autoimmune encephalomyelitis (EAE), seems to be highly influenced by the gut microbiota. Oral antibiotic immunization and consequent depletion of the gut bacteria, before disease induction, resulted in protection from disease development, along with reduction in pro-inflammatory mediators such as IL-17 and an increased Th2-immune response [19]. On the contrary, the intestinal monocolonization of germ-free mice (sterile) with segmented filamentous bacteria (SFB) restored the disease phenotype, along with an increased number of Th17 cells in the CNS, suggesting a direct interplay of the gut microbiota and the immune response in EAE development [20].

Based on these observations, we hypothesized that the gut microbiota itself might play a major role not only in the establishment but also in the reproducibility of the GO animal model described above. The presence or absence not only of pathogens but also of symbiotic and commensal bacteria can favor an immune response more prone to inflammation and conducive to autoimmunity [21].

We aimed to characterize, for the first time, the gut microbiota of the GD/GO models via a combination of metataxonomics (16S rRNA gene sequencing) and traditional microbial culture approaches. We compared the gut contents of TSHR-immunized mice from the two centers to understand whether variation in gut composition could explain differences in the disease induced. Within one center, we then characterized the gut microbiota between different immunizations (TSHR and βgal) and compared them with untreated mice, to determine whether the gut microbiota can influence the outcome and correlate with disease features.

## Methods

### GO preclinical mouse model samples

Mouse samples used in the present work were obtained from a recent study [12], conducted in parallel in two independent animal housing units, under comparable SPF conditions. Animal procedures in center 1 were reviewed and approved by the Ethical Committee of King’s College London and conducted with Personal and Project licenses under United Kingdom Home Office regulations. Animal procedures in center 2 were reviewed and approved by North Rhine Westphalian State Agency for Nature, Environment and Consumer Protection (LANUV), Germany. Samples from the animal unit of King’s College London (UK) will be referred to as “Center 1” and included a total of 5 TSHR-immunized mice (TSHR). Samples from the University of Duisburg-Essen (Germany) will be referred to as “Center 2”, including 10 TSHR-immunized (TSHR), 8 βgal plasmid controls (βgal), and 6 untreated mice (included as a background control), as shown in Table 1.

**Table 1:** Description of the mouse groups involved in this study

No. of animals	Immunization	Centers	Source	Timepoint
5	TSHR	1	Intestinal scraping	T4
10	TSHR	2	I.S./Feces	T0–T4*
8	βgal	2	I.S./Feces	T0–T4*
6	Untreated	2	I.S./Feces	T4°

A total of 23 female BALB/cOlaHsd, 6–8-week-old mice were challenged either with the pTriEx1.1Neo-hTSHR to induce disease (TSHR group) or with pTriEx1.1Neo-β-gal as a plasmid control group (βgal group). Independent SPF animal units were based in London (Center 1) and Essen (Center 2). An untreated group of six mice has been included as a background control. Samples collection comprised of intestine scraping (I.S.) from Center 1 and both fecal pellets and I.S. within Center 2

*Fecal pellets of βgal and TSHR-immunized mice have been collected before any immunization (T0) and during the time course of the immunization protocol until the sacrifice (T4), as represented in Additional file 1: Figure S1

°Untreated mice were sampled at T4 before (fecal) and after the sacrifice (intestinal scraping)

The GO immunization protocol has been previously described [13]. Briefly, 6–8-week-old BALB/cOlaHsd female mice were immunized via intramuscular injection into each biceps femoris muscle [22] and electroporation of either the eukaryotic expression plasmid pTriEx1.1Neo-hTSHR (hTSHR289) (TSHR group) or the control plasmid pTriEx1.1Neo-β-gal (plasmid-control, βgal group). All animals, whether TSHR or βgal controls, received a total of four plasmid injections at 3-week intervals of the experiment (0, 3, 6, and 9 weeks).

Each mouse was anesthetized using isoflurane with a properly calibrated vaporizer throughout the immunization procedure (injection and electroporation). After the immunization, mice were carefully transferred to a recovery cage until fully recovered.

Mice in Center 1 were maintained conventionally in open cages in one room and co-housed at a maximum of three animals per cage. In Center 2, the mice were co-housed according to their immunizations, two to four animals per individually ventilated cage in one room. All mice were provided by different outlets of the same supplier (Harlan Ltd. or Harlan laboratories BV). In both centers, mice received autoclaved water and had been fed ad libitum similar commercial chow from different suppliers (Rat and Mouse no.1 Maintenance from Special Diet Services, LBS Biotech UK for Center 1 and Rat/Mouse Maintenance V1534-300 from Ssniff Spezialadiaten GmbH, Germany, for Center 2). Also the cage bedding was from different suppliers.

All immunized and control mice in both locations were sacrificed 9 weeks after the last immunization (18 weeks) to permit the development of the chronic phase of the disease in the TSHR group (Additional file 1: Figure S1).

After sacrifice, murine intestines were snap-frozen and stored in sterile containers at − 80 °C. For subsequent analysis, whole intestines were thawed and directly afterwards placed on a sterile padding. The organs were dissected into two or three parts and the content of all parts was scratched out from oral to aboral end with a sterile inoculation loop resulting in one sample per mouse, which was collected in a sterile container and frozen at − 80 °C until needed. Within Center 2 only, fecal pellets of βgal- and TSHR-immunized mice were also collected before each injection (week 0, 3, 6, and 9). Total DNA was extracted from fecal pellets as described below.

Methods for (i) the evaluation of clinical symptoms, (ii) the determination of the thyroid hormone thyroxine blood levels (fT4) and TRAB (both stimulating TSAb and blocking TSBAb) antibodies, and (iii) the measurement of the expansion of fat cells (adipogenesis) and muscular atrophy in the orbit have been already described [12]. A full description of the mice involved and samples collected in the present study is represented in Table 1.

### Traditional microbial cultures of mouse gut contents

A total of 29 scraped intestinal samples (Table 1) derived from Center 1 and Center 2 were analyzed. One gram of feces per mouse was diluted in 9 mL pre-reduced maximum recovery diluent (CM0733, Oxoid, Basingstoke, United Kingdom) with 20% *v*/*v* glycerol and the solution was mixed by vortexing for 5 s. Afterwards, 10-fold serial dilutions were prepared, and 100 μl of each dilution was plated onto different culture media under aerobic or anaerobic conditions (Anaerobic Workstation, AW400SG, Elektrotek, Keighley, West Yorkshire, UK). Specific media, culture conditions, and dilution used to isolate different bacteria are listed in Additional file 2.

Bacteria were identified by Gram staining, colony morphology, the presence of spores, and catalase reaction and partially by the API system (BioMerieux, Marcy-l’Étoile, France). Viable bacterial cell counts were enumerated and all counts were recorded as the numbers of log 10 colony forming units per gram of sample. Counts data were Box-Cox transformed before statistical analysis [23]. Mouse groups were compared through the analysis of variance (one-way ANOVA) and Tukey’s multiple comparisons test with adjusted *P* values.

### Extraction of total DNA from gut contents and feces and 16S rRNA gene sequencing

A total of 29 scraped intestinal samples and 96 fecal pellets were individually placed in 2-mL tubes prefilled with 0.1 mm silica and zirconia bead mix (Benchmark Scientific, Edison, USA), dissolved in 1 mL InhibitEX buffer (Qiagen Ltd., West Sussex, UK) and vortexed until homogenized. A bead-beating step (Beadbug microcentrifuge homogenizer, Benchmark Scientific, USA) was applied for 3 × 60 s at 5 m/s with 5 min rest in-between. The DNA extraction has been performed with QiAmp Fast DNA Stool Mini kit (Qiagen Ltd., UK), following the manufacturer’s instruction. Total genomic DNA was eluted in sterile microcentrifuge tubes and quantified by Qubit Fluorimetric Quantitation (ThermoFisher Scientific Ltd., UK), following the manufacturer’s instructions. DNA aliquots were kept at − 20 °C until used. Sequencing of the variable regions of the 16S rRNA gene was performed at Research and Testing Laboratory LLC (Lubbock, Texas, USA). Primers used to amplify the V1–V2 regions of 16S rRNA gene were 28F (5′-GAGTTTGATCNTGGCTCAG-3′) and 388R (5′-TGCTGCCTCCCGTAGGAGT-3′). Sequencing was performed using an Illumina Miseq (Illumina, San Diego, USA), with 10K paired-end sequencing protocol.

### Processing and statistical analysis of metataxonomic data

Processing of the sequences was performed using Mothur v1.36, to reduce possible PCR effects and to cluster sequences into operational taxonomic units (OTUs) at the 97% identity cut-off and provide the taxonomic annotations [24]. Paired-end reads (R1 and R2) were joined for each sample using the Mothur function “make.contigs” and trimmed at the 2.5%-tile and 97.5%-tile on the distribution lengths of the amplicons. Sequences with any ambiguities (i.e., Ns) were removed by setting parameter *N* = 0. Filtered sequences were aligned against the SILVA 16S rRNA gene reference database (http://www.arb-silva.de). Removal of chimera sequences was done with the Uchime tool [25]; singleton and non-bacterial sequences (e.g., *Archaea*, eukaryotic, chloroplast, and mitochondria) have been removed from the analysis. The taxonomic assignment from phylum to genus level of the processed sequences was done using the Ribosomal Database Project (RDP) Naïve Bayesian Classifier, using Trainset 14 with a cut-off of 80% [26]. FastTree (version 2.1.7) has been used to build a phylogenetic tree, using an approximated maximum likelihood solved by Jukes-Cantor evolutionary model [27]. To reduce the effect of possible different sampling methods and to obtain comparable sequencing libraries, each sample library has been subsampled based on the smallest library size. OTUs with less than 10 counts have been excluded from the dataset and grouped as “OTU_low”, and the analysis has been performed collapsing OTUs at the phylum-genus levels. Statistical analysis was performed in R (Version 3.2.2) and STAMP tool for metataxonomic data analysis [28].

Alpha diversity indices (observed OTUs, Chao1, ACE, and Shannon) were calculated within samples from Mothur and tested for association with covariates (e.g., locations and immunizations) using a linear model, followed by Tukey’s honest significant difference (HSD) post hoc analysis.

Beta diversity was estimated using the Unifrac weighted distance to compare bacterial communities among samples [29], and represented in a non-metric dimensional scaling (NMDS) plot. The permutational multivariate analysis of variance (PERMANOVA) was calculated through the Adonis function [30] in R Vegan package (using 999 permutations) and was used to test the association between the microbiota composition and the covariates (e.g., location of the laboratories or immunizations).

The hierarchical clustering of genera was performed using the Spearman distance and the Ward agglomeration method. Statistical tests with *P* ≤ 0.05 were considered as significant.

Over multiple timepoints, the effects of time, immunizations, and their interactions have been estimated on the fecal microbiota composition, all by means of the following linear model:

$$ {y}_{ij k}=\mu +{\mathrm{Time}}_i+{\mathrm{Immunization}}_j+{\left(\mathrm{Time}\times \mathrm{Immunization}\right)}_{ij}+{e}_{ij k} $$

where *y* is the vector of either alpha diversity Chao or Shannon indices, or of the *Firmicutes/Bacteroidetes* ratio calculated from the relative abundances in each sample at each timepoint; *μ* is the overall mean; time is the effect of timepoint in classes (T0, T1…T4); immunization is the type of immunization (either the TSHR or βgal). The factorial interaction between immunization and time has also been included in the model; *e* is the vector of residual effects. Comparison between βgal and TSHR immunizations at each timepoint was made using the pairwise *T* test with Benjamini-Hochberg correction for false discovery rate (FDR).

To test differences in phylum and genus counts between immunizations and timepoints, the same model was used in the generalized linear model (GLM) implemented in the EdgeR package [31]. Pairwise comparisons of phylum and genus counts between timepoints and immunizations have been assessed with Fisher’s exact test in EdgeR package.

Correlations of either the taxonomy counts (phylum and genus relative abundances) or the microbial counts from the traditional culture approach and disease features, such as anti-TSHR antibodies and thyroid hormone thyroxine levels (fT4), orbital adipogenesis, or muscular atrophy values, were estimated using the Spearman correlation coefficient (Rho) and represented in a correlation plot, using the R Corrplot package. Additional statistical methods are described in Additional file 2.

## Results

### Sequencing metrics

From 16S rRNA gene sequencing (V1–V2 regions), a total of 5,333,798 reads were obtained which reduced to 4,047,186 reads after quality filtering. Following alignment, we obtained an average of 20,534 reads per sample, ranging from 3502 to 134,901. Subsampling per library size resulted in a 96% average coverage per OTU definition at 3052 reads per sample. The averaged coverage and subsampling was sufficient to describe gut bacterial communities according to sequence-based rarefaction curves (data not shown).

We identified a total of 4281 OTUs: 1037 OTUs had more than 10 counts across samples and were retained.

### Comparative analysis of the gut microbiota of GO preclinical mouse models in different centers

To assess whether the microbiota has an impact on the GO mouse model in different laboratories, we compared the gut microbial contents of 5 TSHR mice from Center 1 and 10 TSHR-immunized BALB/c female mice from Center 2, after sacrifice (T4).

**Fig. 1:**
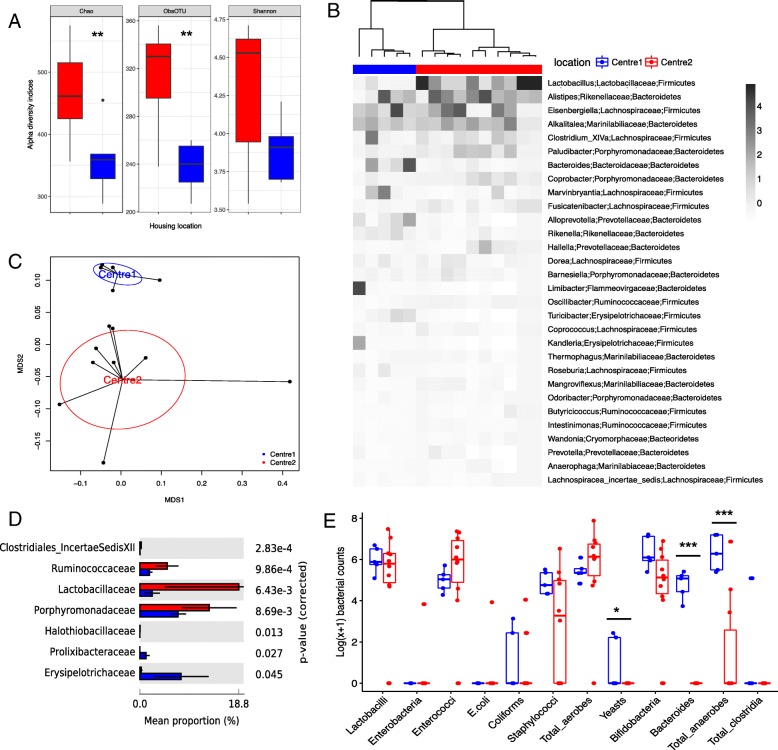
Comparative analysis of the gut microbiota in independent animal units. **a** Box and whisker plot of the alpha diversity indices for richness (Chao1 and observed OTUs indices) and evenness (Shannon index) of the bacterial communities in TSHR-immunized mice housed in Center 1 (blue) and Center 2 (red), respectively. Tukey’s HSD post hoc: Chao1, *P* = 0.01; observed OTUs, *P* < 0.001; Shannon, *P* = 0.08. **b** Annotated heatmap based on Spearman distance and Ward hierarchical clustering of the top 30 genera shows how well the two locations cluster together. Taxonomy explanation includes genera, family, and phylum, which are entered in order of abundance. Genus abundance is described by the change in the intensity of the gray color, as annotated. **c** Multidimensional scaling plot (MDS) based on the weighted Unifrac distances between the two animal units. PERMANOVA with 999 permutations *P* = 0.005. **d** Differentially abundant family from a pairwise comparison with Welch’s *t* test with 95% confidence intervals (STAMP). **e** Box and whisker plot culture results from intestinal scraped samples derived from TSHR-immunized mice from Center 1 and Center 2. Results are expressed as a Log(*x* + 1) transformed colony-forming units/gram feces (cfu/g). *P* values: * *P* < 0.05; ** *P* < 0.001; *** *P* < 0.005

Comparison of the alpha diversity indices showed a significant reduction in the richness (*P* = 0.01), but not in the diversity of the Center 2 microbial community (*P* > 0.05, Fig. 1a). The gut microbiota composition from the two centers showed a good separation according to the Spearman distance and Ward hierarchical clustering (Fig. 1b), and a PERMANOVA test on the weighted Unifrac distances revealed a spatial difference between bacterial communities (*P =* 0.005 with 999 permutations, Fig. 1c).

At a phylum level, *Bacteroidetes* and *Firmicutes* were the most represented of the seven phyla identified, with no differences between them in the two centers (*P =* 0.99). *Lactobacillaceae*, *Ruminococcaceae*, and *Porphyromonadaceae* families were more abundant in Center 2 than in Center 1 TSHR mice (*P* < 0.01, Fig. 1d). We observed significant differences in the abundance of 18 genera between the two centers, as detailed in Table 2.

**Table 2:** Genera differentially abundant between Center 1 (*n* = 5) and Center 2 (*n* = 10) TSHR-immunized mice intestinal scraped samples

Genera	Center 1: mean freq. (%)	Center 2: mean freq. (%)	*P* values
*Allobaculum*	1.001	0.003	0.042
*Alloprevotella*	6.135	0.432	0.003
*Bacteroides*	9.370	1.525	0.017
*Bifidobacterium*	0.668	0.006	0.003
*Clostridium XI*	0.840	0.000	0.005
*Coprobacter*	1.835	4.226	0.033
*Fusicatenibacter*	0.989	3.295	0.032
*Guggenheimella*	0.006	0.169	0.011
*Helicobacter*	0.200	0.000	0.024
*Intestinimonas*	0.097	0.861	0.000
*Lactobacillus*	2.304	18.632	0.030
*Lactonifactor*	0.023	0.401	0.025
*Meniscus*	1.149	0.000	0.000
*Oscillibacter*	0.640	1.748	0.011
*Parabacteroides*	0.292	0.031	0.015
*Pseudoflavonifractor*	0.154	0.466	0.028
*Rikenella*	3.921	1.216	0.004
*Turicibacter*	3.629	0.000	0.002

ANOVA with Tukey’s HSD post hoc analysis (95% confidence interval), generated with STAMP. Mean freq: mean frequency (%)

From the traditional microbial culture of the gut contents, we observed differences in yeast (*P* = 0.03186), *Bacteroides* (*P* < 0.0005), and total anaerobe (*P* = 0.00081) counts, which were found to be enriched in the Center 1 compared with the Center 2 TSHR mice (Table 3). Cultures from mouse intestinal scraping of Center 2 did not contain any total clostridia, *Bacteroides*, or yeasts, and we were able to culture enterobacteria, *E. coli*, and coliforms from only one mouse from this group. *E.coli* and coliforms were also the least abundant in the Center 2 TSHR mice (Fig. 1e). Furthermore, since *Yersinia enterocolitica* has been implicated in GD pathogenesis [32], we used selective agar plates for *Yersinia* sp. but no *Yersinia* colonies grew.

**Table 3:** Traditional microbiology results from TSHR-immunized mouse intestinal scraping from Center 1 (*n* = 5) and Center 2 (*n* = 10)

Microbial target	Center 1: mean counts	Center 2: mean counts	*P* values
*Bacteroides*	1.15E+05	b.d.l.	0.000
Bifidobacteria	6.41E+06	1.32E+06	0.057
Coliforms	3.27E+02	1.15E+03	0.453
*E.coli*	b.d.l.	8.45E+02	0.499
Enterobacteria	b.d.l.	6.82E+02	0.499
Enterococci	1.74E+05	6.10E+06	0.247
Lactobacilli	1.93E+06	4.68E+06	0.725
Staphylococci	1.31E+05	3.77E+05	0.175
Total aerobes	4.18E+05	9.90E+06	0.370
Total anaerobes	6.75E+06	7.39E+05	0.001
Total Clostridia	2.46E+04	b.d.l.	0.165
Yeast	8.72E+01	b.d.l.	0.031

b.d.l.: below detection limit. Detection limits are the following according to the agar used: 1000 CFU/g feces for *Bacteroides*, 100 CFU/g feces for *E.coli* and coliforms as well as for enterobacteria, and 10 CFU/g feces for total clostridia and yeasts, respectively. Microbiological counts were Box-Cox transformed. *P* values obtained by linear regression

### Gut microbiota differences in immunized and control mice within Center 2

**Fig. 2:**
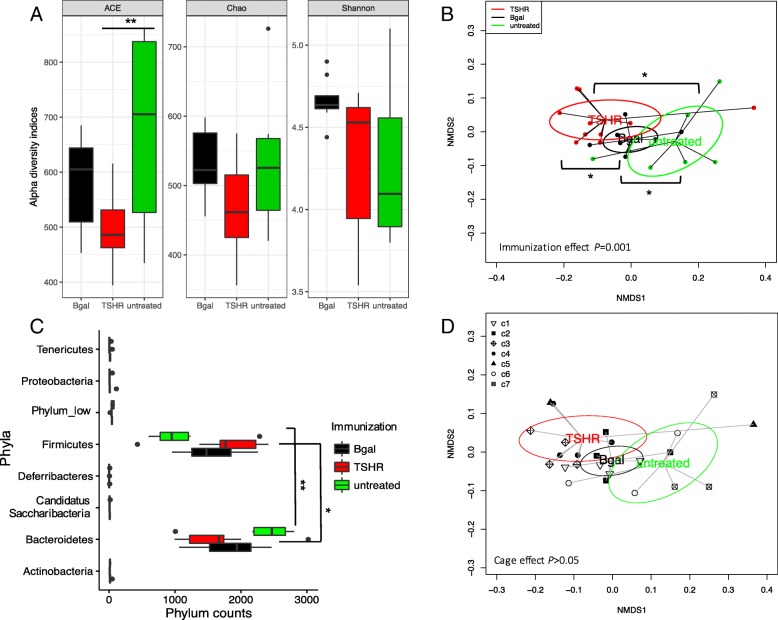
Gut microbiota composition in TSHR-immunized mice and control mice in Center 2 at final timepoint. **a** Box and whisker plots describing the measurement of alpha diversity (Chao, ACE, and Shannon indices). **b** Non-metric dimensional scaling (NMDS) plot of weighted Unifrac distances showed a spatial separation of microbial communities according to the immunizations. PERMANOVA based on 999 permutations *P* = 0.001. **c** Boxplot of the phylum counts according to immunizations. ANOVA on phylum counts BH adjusted *P* < 0.0001 and pairwise *T* test between *Bacteroidetes-Firmicutes* counts adjusted *P* = 0.0003. **d** Non-Metric Dimensional Scaling (NMDS) plot based on weighted Unifrac distances shows spatial separation of the microbial community according to the immunization and caging within Center 2. Mice were co-housed according to their immunization at a maximum of four animals; cages are described by different shapes as in the legend. No significant difference in cage effect is observed. PERMANOVA based on cage effect (999 permutations) for all comparisons *P* = 0.12. *P* values: * *P* ≤ 0.05; ** *P* = 0.01

To observe the possible contribution of the gut microbiota in the disease, we compared the gut microbiota composition between immunization groups in mice within Center 2. No significant differences were observed in alpha diversity indices among immunizations, apart from the abundance-based coverage estimator (ACE) index between untreated and TSHR groups (Fig. 2a, *P* = 0.01). The ACE index relies on the presence of rare OTUs [33], which were more abundant in the untreated group compared to the plasmid-immunized mice. The βgal group showed a slightly skewed distribution of the Shannon index when compared to the others; however, the post hoc comparison was not significant.

The non-metric dimensional scaling (NMDS) of the weighted Unifrac distance matrix showed a separation of the three immunization groups, also confirmed by the permutation test (*P* < 0.01, 999 permutations; Fig. 2b). βgal bacterial communities were closer to those of the untreated mice, while we observed a spatial shift of the TSHR-immunized bacterial communities.

OTUs from *Bacteroidetes* and *Firmicutes* phyla were the most abundant among the phyla identified (Fig. 2c) and showed a different distribution pattern among immunized groups. In particular, *Firmicutes* counts were higher in TSHR-immunized mice (*P* = 0.05) and *Bacteroidetes* were found to be higher in the untreated group (*P* = 0.012). At a genus level, eight genera were differentially abundant between TSHR and βgal groups, three genera between TSHR and the untreated group, and four genera between βgal and the untreated group (Additional file 3: Table S1). We found an enrichment of OTUs of *Acetitomaculum* genus in the βgal group compared to both TSHR (*P* = 0.004) and the untreated group (*P* = 0.003); an enrichment of *Lactobacillus* OTUs in the TSHR compared to the untreated group (*P* = 0.018), and a reduction of *Bacteroides* counts in TSHR when compared to the βgal group (*P* = 0.047). However, no significant differences were observed among immunized groups and in pairwise comparisons generated by the traditional bacterial culturing approach (Additional file 3: Table S2).

In the scraped intestinal samples, we did not observe a cage effect on the composition of the large intestine microbiota (PERMANOVA *P* > 0.05; Fig. 2d).

### Dynamics of fecal microbiota during the immunization protocol

To assess whether the immunization plasmids and the duration of the protocol could have influenced the gut microbiota composition, we sequenced the bacterial 16S rRNA gene from the fecal pellets of the βgal and TSHR group from the baseline (T0) for 18 weeks afterwards, until the end of the experiment (T4).

**Fig. 3:**
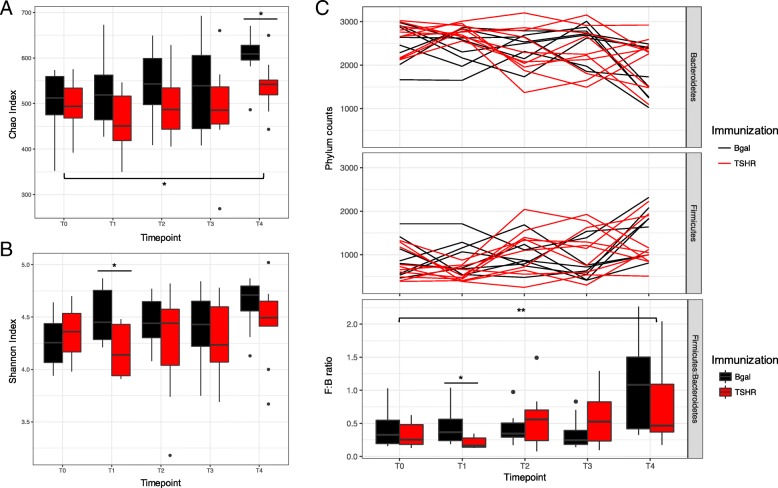
Time-course analysis of GO preclinical fecal microbiota during the immunization protocol. Box and whisker plots of alpha diversity such as Chao (**a**) and Shannon (**b**) indices showed differences over time. **c** Phylum dynamics over time and between immunizations. *Firmicutes* and *Bacteroidetes* were the most abundant phyla, showing differences with time and immunizations. Significant differences among timepoints have been observed at the *Firmicutes/Bacteroidetes* ratio, in particular between the baseline T0 and the last timepoint T4, but not related to immunization. A significant difference in the ratio was observed after 3 weeks from the first injection (T1) between βgal and TSHR. *P* values: * *P* ≤ 0.05; ** *P* = 0.01

We observed a significant increase of the richness (Chao index, Fig. 3a; *P* = 0.02) and the diversity (Shannon index, Fig. 3b) over time, which was less apparent in the TSHR-immunized group. Significant differences regarding richness between TSHR and βgal have been observed at T4 (*P* = 0.027, Table 4). The Shannon index of diversity, on the contrary, was significantly different between TSHR and βgal immunization at T1 (*P* = 0.023, Table 4).

**Table 4:** Summary of the statistics from the time-course analysis of the fecal microbiota during the immunization protocol (T0–T4) and between immunizations (βgal and TSHR)

Index	ANOVA model			TSHR vs. βgal group				
	Immunization	Time	Time × immunization	T0	T1	T2	T3	T4
Chao	0.006	0.02	0.8	0.75	0.066	0.28	0.33	0.027
Shannon	0.054	0.28	0.47	0.44	0.023	0.35	0.35	0.29
Firm:Bact	0.406	0.0003	0.16	0.39	0.028	0.46	0.2	0.26

Firm:Bact, *Firmicutes*/*Bacteroidetes* ratio. ANOVA model as previously described. Pairwise comparison between βgal and TSHR in each time point has been made with a pairwise T-test with Benjamini-Hochberg correction for FDR

**Fig. 4:**
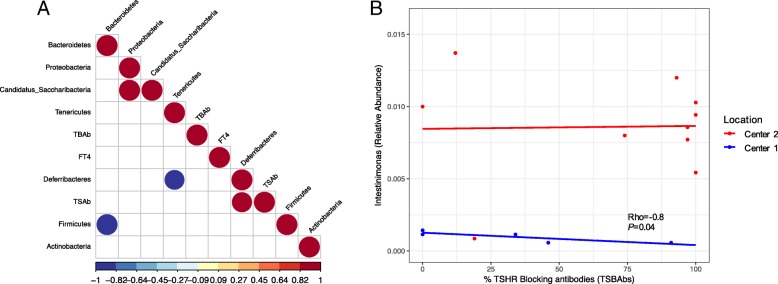
Correlating the gut microbiota and disease features in Center 2 TSHR group. **a** Spearman correlation coefficient strength (Rho) of phylum counts from TSHR mice in Center 2. *Firmicutes* and *Bacteoridetes* showed a strong negative correlation between each other. A positive correlation between the one-genus phylum *Deferribacteres* and the level of thyroid-stimulating antibodies (TSAb) has been observed. Correlations with *P* < 0.05 are shown and strength of the Rho coefficient is represented by the change in the color intensity. fT4, free thyroid hormone thyroxine levels; TSAb, thyroid stimulating antibodies; TSBAb, thyroid-stimulating blocking antibodies (as a percentage values). **b** Enriched *Firmicutes* genus *Intestinimonas* between Center 1 (blue) and Center 2 (red) showed a strong negative correlation with the percentage of thyroid-stimulating blocking antibodies (TSBAbs) at 95% confidence interval in Center 1 (Rho = − 0.8, *P* = 0.04), but not in Center 2

The murine fecal microbiota comprised *Bacteroidetes* and *Firmicutes* phyla predominantly (Fig. 4c); followed by *Tenericutes*, *Proteobacteria Deferribacteres*, and Candidatus Saccharibacteria phyla. The *Firmicutes*/*Bacteroidetes* ratio has been used to describe the shift in the gut microbiota associated with aging [34] and also in disease conditions such as obesity [35]. The ratio showed differences among the timepoints of the experimental procedure (*P* < 0.01) and between TSHR and the βgal group after 3 weeks from the first injection (T1, *P* = 0.011; Fig. 3c).

We fitted a generalized linear model (GLM) to compare the taxonomic counts at different timepoints within each group independently (either TSHR or βgal). Thirty-four genera have been identified as differentially abundant among all timepoints in the TSHR-immunized group (Additional file 4: Table S1), while 25 were found in the βgal group (Additional file 4: Table S2). We observed differences in the taxonomic profile between TSHR and βgal groups at each timepoint using an exact test (EdgeR). Once again, T1 was identified as the timepoint with the highest number of genera differentially expressed, as illustrated by the diversity indices (Additional file 4: Table S3).

In contrast to data obtained from the gut microbiota (Fig. 2d), a cage effect was observed in the fecal microbiota, in particular, in interaction with time (*P* = 0.001) and immunization (*P* = 0.002; Additional file 5: Figure S1). The latter is probably due to the mice being caged according to the type of plasmid injection they received, but we also observed a significant difference within the same immunization group (e.g., TSHR in cage 4 and cage 5, *P* = 0.01).

### Correlating the gut microbiota composition with clinical features and differences in GO development

We then investigated possible correlations between disease features, such as anti-TSHR antibodies, thyroxine levels (fT4), orbital adipogenesis, and muscular atrophy, and the gut microbiota composition to determine whether it contributes to the heterogeneity of induced responses, summarized in Additional file 1: Table S1.

Within the Center 1 TSHR-immunized group, we found that OTUs from *Firmicutes* and *Bacteroidetes* negatively correlated to each other (Rho = − 1, *P* < 0.0001). A positive correlation between levels of TSAb and *Deferribacteres* phylum, which include one-genus *Mucispirillum*, was found (Rho = 0.92, *P* = 0.028; Fig. 4a).

From those genera differentially abundant between TSHR-immunized mice from Center 1 and Center 2 (Table 2), identified via metataxonomics, we observed a strong negative correlation of the *Firmicutes* genus *Intestinimonas* and the levels of TSBAb in the Center 1 (Rho = − 0.89, *P* < 0.05) but not in the Center 2 counterpart (Fig. 4b). No significant correlation was observed between OTUs from the genus *Intestinimonas* and levels of TSAb or levels of free thyroxine hormone (fT4; data not shown).

On the contrary, the Box-Cox transformed counts from the traditional microbiology did not show any significant correlation with the disease features described (data not shown).

**Fig. 5:**
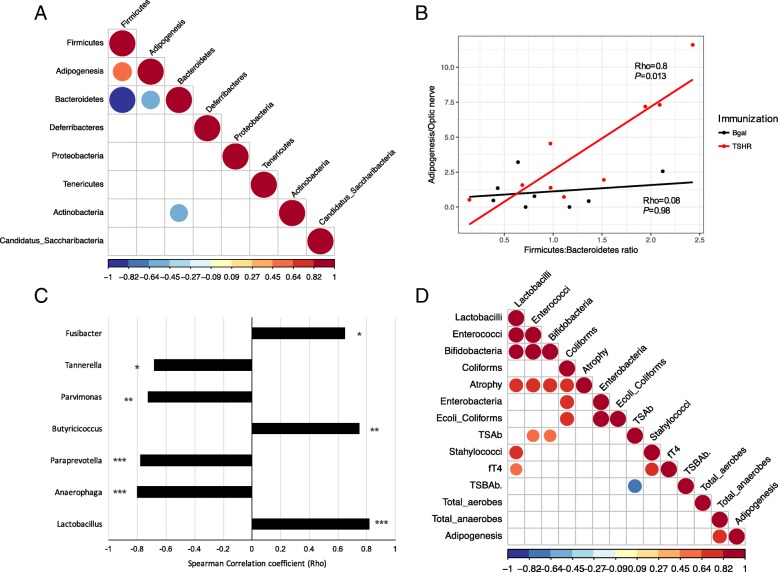
Correlation of the gut microbiota composition with clinical features and differences in Center 2 mice. **a** Correlation plot of phyla and the orbital adipogenesis value. Spearman correlation coefficient strength (Rho) as indicated by the colored bar. Firmicutes and *Bacteoridetes* showed a strong negative correlation between each other. A positive correlation between *Firmicutes* and a negative correlation with *Bacteroidetes* OTUs and the adipogenesis value (calculated in the orbit) has been observed. Adipogenesis clustered closer to the Firmicutes and Bacteroidetes value according to the complete linkage method for hierarchical clustering. Only *P* < 0.05 are shown. **b** Positive strong correlation of the *Firmicutes/Bacteroidetes* ratio with the adipogenesis value (calculated in the orbit) resulted significant in TSHR-immunized group but not in the βgal group. **c** Spearman correlation coefficient (Rho) of genera among phyla *Bacteroidetes* and *Firmicutes* and the orbital adipogenesis values. The strength of the correlation coefficient is represented on *x*-axis: bars on the left represent a negative correlation coefficient, while bars on the right represent a positive correlation coefficient. Correlations with *P* < 0.05 are shown; order of entrance depends on their *P* values: * *P* < 0.05; ** *P* < 0.1; *** *P* < 0.005. **d** Spearman correlation coefficient plot of the Box-Cox transformed microbiological counts and disease features in Center 2 TSHR-immunized mice. Feature clustering was according to the complete linkage method for hierarchical clustering. Only correlations with *P* < 0.05 are shown and strength of the correlation coefficient is represented by the change in the color intensity. fT4, free thyroid hormone thyroxine levels; TSAb, thyroid-stimulating antibodies; TSBAb, thyroid-stimulating blocking antibodies (as a percentage values)

Within Center 2, *Bacteroidetes* and *Firmicutes* negatively correlated to each other (Rho = − 0.99, *P* < 0.0001; Fig. 5a). We also found a significant positive correlation (Rho = 0.6, *P* = 0.009) between the OTUs from the *Firmicutes* and the orbital adipogenesis value and a negative correlation of this value with the phylum *Bacteroidetes* (Rho = − 0.57, *P* = 0.014). As expected, these correlations were specific to the TSHR-immunized mice (Fig. 5b). The correlation pattern we found (*Firmicutes* positively correlated, *Bacteroidetes* negatively correlated) was also recapitulated at the genus level. Among the genera of the *Firmicutes*, three within the Clostridia family (*Butyricicoccus*, *Parvimonas* and *Fusibacter*) and the genus *Lactobacillus* were correlated positively with adipogenesis, while three *Bacteroidetes* genera (*Anaerophaga*, *Paraprevotella*, and *Tannerella*) correlated negatively with the orbital adipogenesis values (Fig. 5c).

A strong positive correlation (Rho = 0.82, *P* = 0.007) was observed between orbital adipogenesis and the total anaerobes counts obtained from the traditional microbial cultures of TSHR-immunized mice, but not in the controls (Fig. 5d). Moreover, from the traditional microbial cultures data, we observed correlations with other disease features, specifically in the TSHR group. We observed strong positive correlations between the muscular atrophy values and the cluster of lactobacilli (Rho = 0.74, *P* = 0.03), enterococci (Rho = 0.8, *P* = 0.02), bifidobacteria (Rho = 0.76, *P* = 0.03), and coliforms (Rho = 0.73, *P* = 0.04). Levels of free thyroxine (fT4) were positively correlated with lactobacilli (Rho = 0.64, *P* = 0.05) and staphylococci (Rho = 0.77, *P* = 0.016).

## Discussion

Animal models have been invaluable in dissecting the mechanisms causing loss of immune tolerance leading to autoimmune conditions such as GD. Thus, we aimed to test the hypothesis that the gut microbiota may affect both outcome and reproducibility of induced autoimmune disease, such as reported in the recent research article of Berchner-Pfannschmidt and co-workers [12].

We observed significant differences in the diversity and spatial organization of the gut microbiota of female TSHR-immunized BALBc mice in two independent SPF units. We also demonstrated disease-associated microbial taxonomies and correlation with ocular disease, suggesting that the gut microbiota have contributed to the heterogeneity of induced response in the two locations, which further supports our hypothesis.

Animals were maintained in similar conditions. We are confident that there were no infections ongoing at the moment of sampling, since animals in both centers were routinely tested for the presence of viruses, mycoplasma and parasites (see Additional file 1: Table S2); moreover, housing facilities had comparable SPF conditions. Animals were from the same supplier but in different countries (Harlan Ltd. for Center 1 and Harlan Lab. BV for Center 2), had received autoclaved water, and had been fed similar commercial chow, with the exception that food pellets provided in Center 2 contained twice the amount of iodide compared to Center 1 food (see Additional file 1: Table S3). Although iodide excess can be associated with abnormal thyroid function, we do not consider that this dietary variation is enough to explain the results (i.e., elevated thyroxine levels were apparent in the Center 1 but not in the Center 2 mice). The effect of iodine has been studied in the NOD mouse which spontaneously develops autoimmune thyroiditis. Vecchiatti and colleagues [36] reported that excess iodine (0.2 mg/mouse/day) increased the incidence and severity of disease; however, the BALB/c mice in our study did not display thyroiditis. A transgenic NOD mouse expressing the human TSHR-A subunit is able to develop antibodies to the human TSHR and this too is exacerbated by iodine excess [37] but at levels far greater than in the chow used in Centers 1 and 2. We also considered whether iodine could affect the gut microbiota, in view of its use as an antiseptic, but all the studies we found were in this context, rather than the effect of dietary iodine on symbionts. The importance of SPF conditions is indicated by a previous study which failed to reproduce a GO animal model, despite using mice from the same supplier and identical bedding, water, and chow [10]. However, even SPF may be inadequate since differences were found in the gut microbiota of C57BL/6 colonies bred in two different rooms of the same SPF facility [38], fortunately mice in our study were all housed in the same room.

Cage effects were apparent in the fecal microbiota results, which highlight the importance of studying the gut microbiota instead when comparing autoantigen (TSHR)-immunized and control mice, which are in the close proximity of the intestinal mucosa and the immune system, enabling us to explore its relationship with disease features.

We observed several disease-associated taxonomies; the abundance of the newly described butyrate-producing genus *Intestinimonas* [39] was reduced in the Center 1 group compared to Center 2 and correlated negatively with TSBAb. The *Intestinimonas species butyroproducens* has a unique ability to produce butyrate from lysine and is involved in the detoxification of advanced glycosylation end (AGE) products such as fructoselysin, which have been linked to type 1 diabetes [40], although we are unaware of any link between butyrate-producing bacteria and thyroid autoimmunity.

The TSHR-immunized group developed some signs of GO and their gut microbiota had increased OTUs of the phylum *Firmicutes* but decreased *Bacteroidetes* compared with controls. This mirrors our preliminary data in human disease where we observed a dramatic reduction in the *Bacteroides* genus in GD patients when they develop GO (INDIGO publishable summary^1^).

We also obtained a positive correlation between several *Firmicutes* counts, such as *Clostridia* and *Bacilli*, with orbital adipogenesis in Center 2 TSHR-immunized mice. Million and co-workers have previously reported a positive correlation between OTUs from the *Firmicutes* and weight gain/obesity in both animal models and humans [41]. Interestingly, the role of the genus *Lactobacillus* and its products in either triggering or protecting from adipogenesis has been debated and seems to be species-specific.

In the present work, we could exclude a possible gain-of-weight relationship with the adipogenesis value calculated in the orbit since no changes in mouse weights have been observed during the development of the chronic phase of the disease (data not shown). Furthermore, molecular mechanisms driving obesity and orbital adipogenesis may well be different, since the latter is derived from the neural crest and the gut microbiota may have varying effects on different fat depots [42].

Our time-course analysis revealed that time had a dramatic role in shaping the fecal microbiota of the female mice which were 6–8 weeks old at the outset and 24–26 weeks at the end of the experiment, confirming the work of McCafferty and colleagues [43]. The richness and diversity of βgal control mice increased with age but this was less apparent in TSHR-immunized animals. Significant differences in microbiota composition between control and TSHR immunizations were most apparent 3 weeks after the first immunization, at the initiation of the induced immune response.

Our control group comprised mice immunized with the βgal expression plasmid in which we observed a slight skew in the microbiota richness and diversity which may be caused by the systemic overexpression of the β-galactosidase enzyme, whose natural role is in glycan metabolism, e.g., the hydrolysis of the lactose to galactose and glucose [44]. Kaneda and collaborators reported a βgal overexpression peak in the muscle fibers following electroporation from 5 days to 2 weeks after the injection [45].

It may be that the increased OTUs of the *Firmicutes* genus *Acetitomaculum* was specifically triggered by the product of the βgal enzymatic reaction over time (Additional file 4: Table S2). This effect merits further investigation but we are confident that the βgal vector plasmid provides the optimum control group since its microbial communities were more closely related to that of the naïve non-immunized group than to TSHR-immunized mice. Of interest, TSHR-immunized mice in Center 2 were more similar to TSHR-immunized mice from Center 1 (*P* = 0.2) than βgal (*P* = 0.024), than untreated (*P* = 0.04) mice in their own center (Additional file 6: Figure S1).

The results we obtained using 16S rRNA gene metataxonomics and via the traditional microbial culture approach were largely similar, with relatively few differences. Microbial cultures revealed significantly higher yeast counts (*P* = 0.03186) in Center 2 TSHR-immunized mice—which obviously could not be seen via the bacterial metataxonomics—and a nearly significant difference in the *Actinobacteria* genus *Bifidobacterium* (*P* = 0.057), which was not detected in our metataxonomics data. Primers based on the V1–V2 regions of the 16S rRNA gene did not detect *Bifidobacterium* OTUs. Consequently, we applied a new set of primers (28F-combo) with which we observed a significant enrichment of bifidobacteria counts in Center 2 (Additional file 7: Figure S1), in agreement with the microbial culture results.

## Conclusions

In conclusion, our results indicate a role for the gut microbiota in modulating the heterogeneity apparent in the TSHR-induced model of GD and GO. In our next study, we will report the effects on our induced model of modifying the gut microbiota using antibiotics, probiotics, and fecal material transfer.

Our future studies will investigate whether the presence, absence, or amounts of certain bacteria or yeast have the ability to directly alter the immune balance between the Treg anti-inflammatory response and the Th17-mediated pro-inflammatory response in the gut mucosa as has been reported in models of other autoimmune diseases [22, 46]. Results of these experiments could then be confirmed by colonization studies in gnotobiotic animals. Factors such as level of dietary iodine intake and age of mice at immunization, which may both alter the gut microbiota and/or immune responsiveness, are also warranted.

## Additional files

Additional file 1: **Figure S1.** Schematic representation of the GO immunization protocol and sample collection. **Table S1.** Summary of disease characteristics induced in mice in Center 1 and Center 2 using TSHR expression plasmid illustrating the heterogeneity of response. **Table S2.** Quarterly Health Screen Reports on viral, bacterial, mycoplasma and parasite screen in both centers. **Table S3.** Composition of the commercial chows provided ad libitum in Center 1 and Center 2. (DOCX 106 kb)

Additional file 2: Supplementary methods. (DOCX 121 kb)

Additional file 3: **Table S1.** Differential abundant taxonomic analysis between TSHR (*n* = 10), βgal (*n* = 8), and untreated (*n* = 6), within Center 2. Welch’s T-test with 95% confidence interval using STAMP. Mean relative frequency, rel. freq. Standard deviation, std. dev. **Table S2.** Comparison of intestinal scraped samples from different immunization within Center 2 from the traditional microbiological culture. Data were Box-Cox transformed. (XLSX 43 kb)

Additional file 4: **Table S1.** Generalized linear model (GLM) of genera counts differentially present in TSHR-immunized mice over timepoints, in reference to the baseline (T0) using EdgeR. LogFC, Log2 fold change between each timepoint and the baseline (T0); LR, likelihood ratio. **Table S2.** Generalized linear model (GLM) of genera counts in βgal control mice over timepoints using EdgeR. LogFC, Log2 fold change between each timepoint and the baseline (T0); LR, likelihood ratio. **Table S3.** Pairwise comparison of TSHR and βgal mice using Fisher’s Exact Test in EdgeR at each timepoint (T0 to T4). LogFC, Log2 fold change of βgal compared to TSHR. (XLSX 46 kb)

Additional file 5: **Figure S1**. Temporal stability of fecal microbiota and cage effect of the immunizations. Weighted Unifrac distances of mice fecal microbial communities represented over the time course of the experiment according to the immunization (A) or the cage (B). Permutational MANOVA of weighted Unifrac distances according to timepoint, immunizations, caging, and their interactions (time × cage; time × immunization; immunization × cage) as described in Additional file 2. The time had a significant effect on the stability of the fecal microbiota (*P* = 0.001), in particular between the baseline (T0) and the last timepoint (T4, *P* = 0.003); and between the T1 and T4 (*P* = 0.009). The interaction between time and immunization was significant (*P* = 0.007). Cage was also significant, in particular the interaction cage × timepoint (*P* = 0.001) and cage × immunization (*P* = 0.002). Significant differences within the same immunization group cage has been observed (TSHR group in C4 and C5, *P* = 0.01). (PDF 152 kb)

Additional file 6: **Figure S1.** NMDS plot based on the weighted Unifrac distances of Center2 immune and control mice including TSHR-immunized mice from Center 1. TSHR-immunized mice from Center 1 were more similar to TSHR-immunized mice from Center 2 (*P* = 0.2) than to the βgal (*P* = 0.024) than the untreated (*P* = 0.04). (PDF 28 kb)

Additional file 7: **Figure S1.** *Bifidobacterium* counts derived from the 28F-combo primers in the TSHR-immunized mice in Center 1 (*n* = 5) and Center 2 (*n* = 10). ANOVA with Tukey’s HSD post hoc analysis (95% confidence interval), *P* value = 0.003 generated with STAMP. (PDF 21 kb)

## Abbreviations

ACE: Abundance-based coverage estimator
AGE: Advanced glycosylation end
CNS: Central nervous system
EAE: Experimental autoimmune encephalomyelitis
FDR: False discovery rate
fT4: Thyroid hormone thyroxine
GD: Graves’ disease
GLM: Generalized linear model
GO: Graves’ orbitopathy or ophthalmopathy
HSD: Honest significant difference
NMDS: Non-metric dimensional scaling
OTU: Operational taxonomic unit
PERMANOVA: Permutational multivariate analysis of variance
RDP: Ribosomal Database Project
SFB: Segmented filamentous bacteria
SPF: Specific-pathogen-free
TRAB: Thyroid-stimulating hormone autoantibodies
Treg: Regulatory T cells
TSAb: Thyroid-stimulating antibodies
TSBAb: Thyroid-stimulating blocking antibodies
TSH: Thyroid-stimulating hormone
TSHR: Thyrotropin receptor
βgal: β-Galactosidase enzyme

## Footnotes

1 INDIGO publishable summary: http://www.indigo-iapp.eu/publishable-summary/
