# Supplementary material for: Gut microbiota in experimental murine model of Graves’ orbitopathy established in different environments may modulate clinical presentation of disease
Source: Microbiome. 2018 May 25;6:97. doi: 10.1186/s40168-018-0478-4 (PMC5970527; doi:10.1186/s40168-018-0478-4)
Supplement: Supplementary file 1 — Figure S1. Schematic representation of the GO immunization protocol and sample collection. Table S1. Summary of disease characteristics induced in mice in Center 1 and Center 2 using TSHR expression plasmid illustrating the heterogeneity of response. Table S2. Quarterly Health Screen Reports on viral, bacterial, mycoplasma and parasite screen in both centers. Table S3. Composition of the commercial chows provided ad libitum in Center 1 and Center 2. (DOCX 106 kb) [file 40168_2018_478_MOESM1_ESM.docx]

**Additional file 1**

**Figure S1**


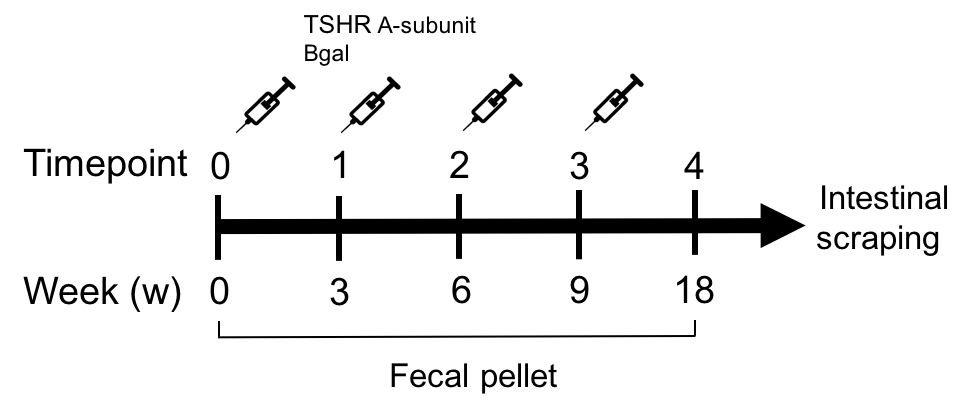


**Schematic representation of the GO immunization protocol and sample collection**. Female BALB/cOlaHsd, 6-8 weeks old mice were immunized via intramuscular injection and electroporation of either the eukaryotic expression plasmid pTriEx1.1Neo-hTSHR (hTSHR289) to develop signs of GO (TSHR A-subunit) or the control plasmid pTriEx1.1Neo-β-gal, as a plasmid-control group (βgal). Each animal received a total of four plasmid injections at three week-intervals. All immunized and control mice were sacrificed 9 weeks after the last immunization to permit the development of the chronic phase of the disease in the TSHR immunized group. Fecal pellets have been collected during the time course of the immunization trial from the baseline (T0) and before any other injection until the end of the procedure (T4). After euthanasia, the microbial content residing on the colonic mucosa has been collected through scraping.

**Table S1** Summary of disease characteristics induced in mice in Center 1 and Center 2 using TSHR expression plasmid illustrating the heterogeneity of response.

| **Disease Feature** | **Center 1 (n=5)** | **Center 2 (n=10)** |
| --- | --- | --- |
| TRAB (%) | 5/5 positive | 10/10 positive |
| TSAb (pmol/mL) | 2/5 positive | 4/10 positive |
| TSBAb (%) | 3/5 positive | 9/10 positive |
| Thyroxine (mg/dL) | 2/5 hyperthyroid | 10/10 euthyroid |
| Orbital adipogenesis | Not available | 4/8 increased |
| Orbital muscle atrophy | Not available | 3/8 significantly increased |
| Thyroid Histology | 2/5 thyroid focal infiltration | 10/10 normal histology |

**Table S2** Quarterly Health Screen Reports on viral, bacterial, mycoplasma and parasite screen in both centers.

| **Microbiological screening** | **Center 1** | **Center 2** |
| --- | --- | --- |
| MNV (Murine Norovirus) | negative | positive |
| Helicobacter | negative | positive |
| Trichomonas sp. | negative | positive |

**Table S3** Composition of the commercial chows provided *ad libitum* in Center1 and Center2

|  | **Center 1** | **Center 2** |
| --- | --- | --- |
| Product Name | Rat and Mouse no.1 Maintenance | Rat/Mouse Maintenance V1534-300 |
| Supplier | Special Diet Services, LBS Biotech UK | Ssniff Spezialadiaten GmbH, Germany |
| Protein (%) | 14.38 | 19.00 |
| Fat (%) | 2.71 | 3.30 |
| Fiber (%) | 4.65 | 4.90 |
| Ca (%) | 0.73 | 1.10 |
| P (%) | 0.52 | 0.70 |
| Na (%) | 0.25 | 0.24 |
| I (mg/Kg) | 1.2 | 2.2 |
| Gross energy (MJ/Kg) | 14.74 | 16.3 |
| Metabolizable energy (MJ/Kg) | 10.74 | 12.8 |
