# Supplementary material for: Gut microbiota in experimental murine model of Graves’ orbitopathy established in different environments may modulate clinical presentation of disease
Source: Microbiome. 2018 May 25;6:97. doi: 10.1186/s40168-018-0478-4 (PMC5970527; doi:10.1186/s40168-018-0478-4)
Supplement: Supplementary file 2 — Supplementary methods. (DOCX 121 kb) [file 40168_2018_478_MOESM2_ESM.docx]

**Additional file 2**

**Supplementary methods**

### **Media and conditions for microbial cultures**

The following media, culture conditions and dilutions were used to isolate different bacteria in this study: Horse blood agar (Horse blood agar base No.2 (CM0271) with added Defibrinated Horse Blood (SR0050); both Oxoid, Basingstoke, United Kingdom) was incubated under aerobic conditions for 24 hours at 36 +/- 1 °C. Dilutions from 10^-2^ to 10^-7^ were made and total cell count was measured. MacConkey agar No. 3 (CM0115, Oxoid, Basingstoke, United Kingdom) was incubated under aerobic conditions for 24 hours at 36 +/- 1 °C. Dilutions from 10^-2^ to 10^-7^ were made and *Enterobacteriaceae (*red colonies with bile precipitation and straw coloured colonies) were counted. Slanetz and Bartley Medium (CM0377, Oxoid, Basingstoke, United Kingdom) was incubated under aerobic conditions for 24 hours at 36 +/- 1 °C. Dilutions from 10^-2^ to 10^-7^ were prepared and enterococci (deep red coloured colonies) were counted. Brilliance *E. coli* / coliform selective agar (CM1046, Oxoid, Basingstoke, United Kingdom) was incubated under aerobic conditions for 24 hours at 36 +/- 1 °C. Dilutions from 10^-2^ to 10^-7^ were made and *E. coli* (purple colonies) were counted. Tergitol 7 agar (CM0793, Oxoid, Basingstoke, United Kingdom) was incubated under aerobic conditions for 24 hours at 36 +/- 1 °C. Dilutions from 10^-2^ to 10^-7^ were prepared and coliforms (any color) were counted. Baird Parker agar base (CM0275, Oxoid, Basingstoke, United Kingdom) with added 50 ml of Egg Yolk Tellurite Emulsion (SR0054, Oxoid, Basingstoke, United Kingdom) was incubated under aerobic conditions for 48 hours at 36 +/- 1 °C. Dilutions from 10^-2^ to 10^-7^ were made and *Staphylococcus aureus* (black, shiny colonies with white and clear zones) were counted. Anaerobe basal agar (CM0972, Oxoid, Basingstoke, United Kingdom) was pre-reduced and incubated under anaerobic conditions for 48 hours at 36 +/- 1 °C. Dilutions from 10^-2^ to 10^-7^ were prepared and total cell count was measured. Each colony was checked for aerobic growth and ignored if so. Dichloran Rose-Bengal Chloramphenicol Agar (DRBC agar) (CM0727, Oxoid, Basingstoke, United Kingdom) was incubated under aerobic conditions for 48 hours at 36 +/- 1 °C. Dilutions from 10^-1^ to 10^-2^ were made and total yeast cell count was measured. Wilkins-Chalgren anaerobe agar (Code: CM0619, Oxoid, Basingstoke, United Kingdom) with added 1 vial of G-N Anaerobe Supplement (SR0108) and 25 ml defibrinated blood (SR0050/SR0051, both Oxoid, Basingstoke, United Kingdom) was pre-reduced and incubated under anaerobic conditions for 48 hours at 36 +/- 1 °C. Dilutions from 10^-3^ to 10^-8^ were made and *Bacteroides* spp. (grey/white colonies partially mucoid and with tattered edges) were counted. Each colony was checked for aerobic growth and ignored if so. MRS agar (CM0361, Oxoid, Basingstoke, United Kingdom) with added 1 vial of polymyxin B supplement (SR0099, Oxoid, Basingstoke, United Kingdom) was pre-reduced and incubated under anaerobic conditions for 48 hours at 36 +/- 1 °C. Dilutions from 10^-3^ to 10^-8^ were prepared and lactobacilli (pale straw coloured colonies) were measured. MRS-X agar (see MRS agar added 0.25 g L-cysteine hydrochloride monohydrate, 1 g Lithium chloride and 1.5 g Sodium propionate dissolved in 500 ml deionised water) was pre-reduced and incubated under anaerobic conditions for 48 hours at 36 +/- 1 °C. Dilutions from 10^-3^ to 10^-8^ were made and checked for bifidobacteria (small, shiny colonies). Alcohol shock anaerobe basal agar was prepared as follows: 1 ml of the 1 g faeces diluted in 9 ml maximum recovery diluent with glycerol was mixed with 1 ml ethanol (≥98%, Sigma Aldrich, St. Louis, Missouri, USA) and rolled for 30 min. Conditions were similar to anaerobe basal agar. Dilutions from 10^-1^ to 10^-4^ were made and total cell count was measured. Each colony was checked for aerobic growth and ignored if so.

### **Stability of the faecal microbiota over time**

The function Adonis [Anderson, 2001] implemented in the Vegan package was used to test the variations between-samples of the microbial communities (calculated using the weighted Unifrac distance) over timepoints and among cages, via a permutational analysis of variance or non-parametric MANOVA. The linear predictors and response matrix were as following:

$$y_{ijk}=\mu+T_{i}+ I_{j}+({T*I)}_{ij}+C_{k} +{(C*T)}_{ki} + {(C*I)}_{kj} +e_{ijk}$$

whereas:

$y_{ijk}$ is the weighted Unifrac matrix for treatment *i*, time *j* and cage *k*,

*µ* is the overall mean;

$T_{i}$ is the effect of the *i*th time which was set as a class (T0, T1…T4);

$I_{j}$ is the type of *j*th immunization which is represented by either TSHR or βgal;

$C_{k}$ is the effect of *k*th cage which is expressed as a class (C1, C2…C5);

$({TI)}_{ij} {(CT)}_{ki}$ and ${(CI)}_{kj}$ represent factorial interactions between time, immunizarions and cage;

$e_{ijk}$ is the vector of the residual effects.

A pairwise interaction within immunizations, cages and timepoints has been assessed using a built-in pairwise PERMANOVA script in R.
