# Supplementary figures and images for: Gut microbiota in experimental murine model of Graves’ orbitopathy established in different environments may modulate clinical presentation of disease
Source: Microbiome. 2018 May 25;6:97. doi: 10.1186/s40168-018-0478-4 (PMC5970527; doi:10.1186/s40168-018-0478-4)

# Additional file 5: Figure S1

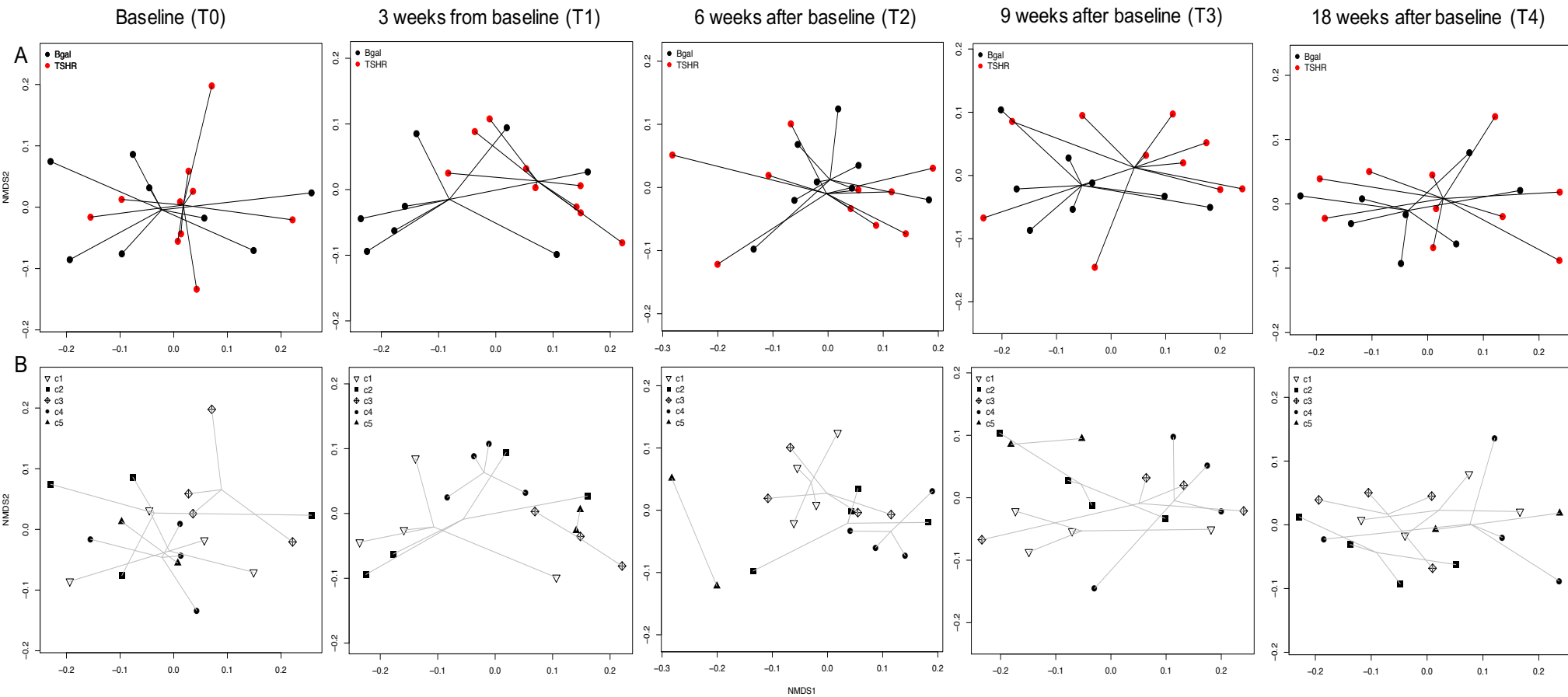

Supplement: Supplementary file 5 — Figure S1. Temporal stability of fecal microbiota and cage effect of the immunizations. Weighted Unifrac distances of mice fecal microbial communities represented over the time course of the experiment according to the immunization (A) or the cage (B). Permutational MANOVA of weighted Unifrac distances according to timepoint, immunizations, caging, and their interactions (time × cage; time × immunization; immunization × cage) as described in Additional file 2. The time had a significant effect on the stability of the fecal microbiota (P = 0.001), in particular between the baseline (T0) and the last timepoint (T4, P = 0.003); and between the T1 and T4 (P = 0.009). The interaction between time and immunization was significant (P = 0.007). Cage was also significant, in particular the interaction cage × timepoint (P = 0.001) and cage × immunization (P = 0.002). Significant differences within the same immunization group cage has been observed (TSHR group in C4 and C5, P = 0.01). (PDF 152 kb) [file 40168_2018_478_MOESM5_ESM.pdf]

## Additional file 6: Figure S1

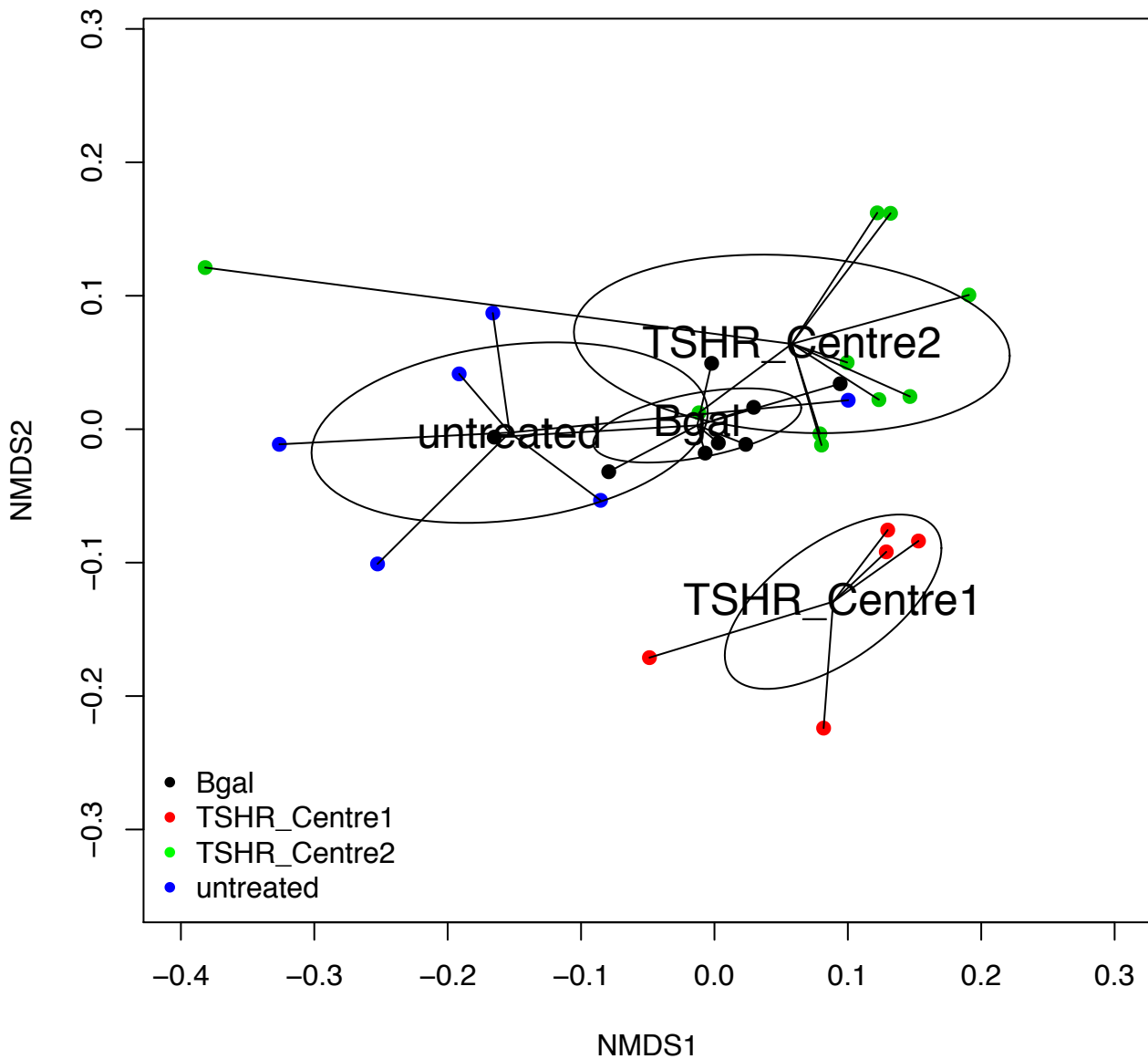

Supplement: Supplementary file 6 — Figure S1. NMDS plot based on the weighted Unifrac distances of Center2 immune and control mice including TSHR-immunized mice from Center 1. TSHR-immunized mice from Center 1 were more similar to TSHR-immunized mice from Center 2 (P = 0.2) than to the βgal (P = 0.024) than the untreated (P = 0.04). (PDF 28 kb) [file 40168_2018_478_MOESM6_ESM.pdf]

## Additional file 7: Figure S1

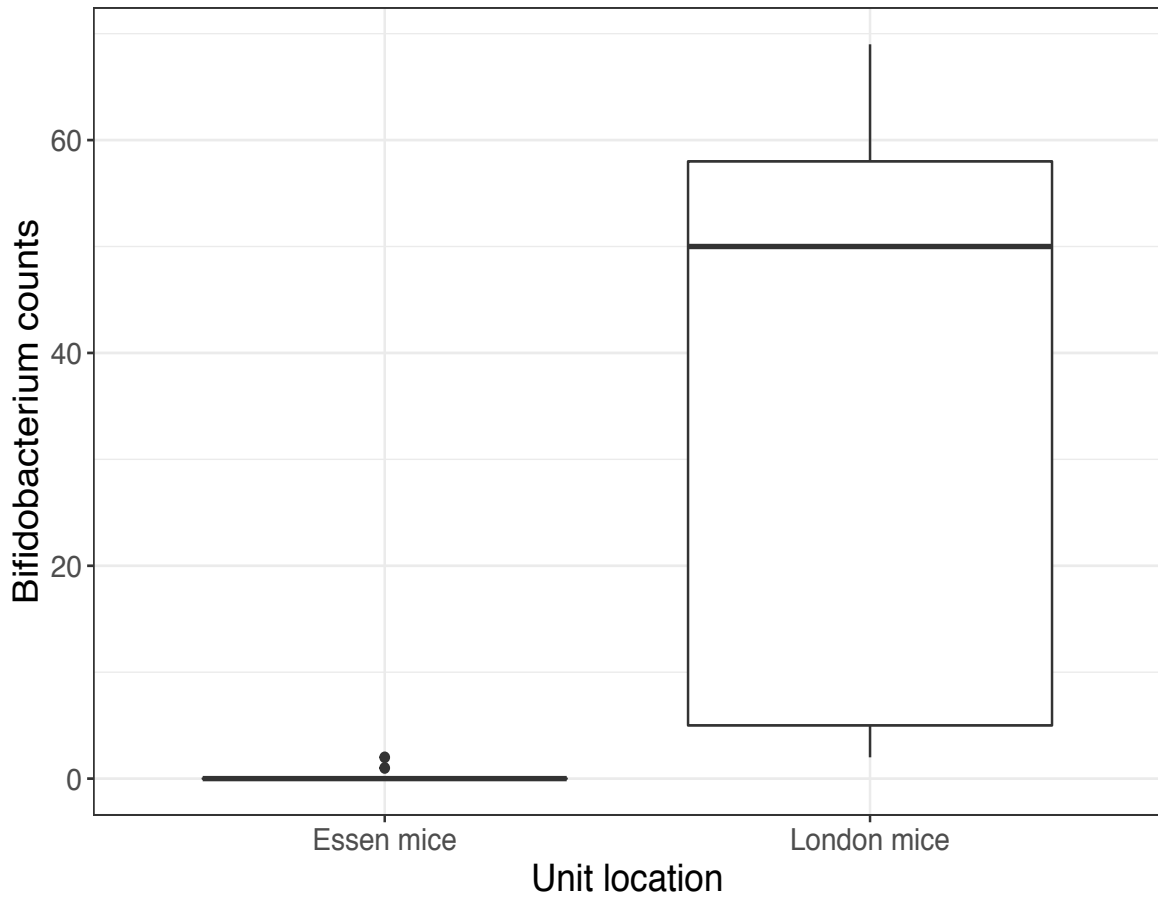

Supplement: Supplementary file 7 — Figure S1. Bifidobacterium counts derived from the 28F-combo primers in the TSHR-immunized mice in Center 1 (n = 5) and Center 2 (n = 10). ANOVA with Tukey’s HSD post hoc analysis (95% confidence interval), P value = 0.003 generated with STAMP. (PDF 21 kb) [file 40168_2018_478_MOESM7_ESM.pdf]
